# The effect of sodium thiosulfate on immune cell metabolism during porcine hemorrhage and resuscitation

**DOI:** 10.3389/fimmu.2023.1125594

**Published:** 2023-02-23

**Authors:** Eva-Maria Wolfschmitt, Melanie Hogg, Josef Albert Vogt, Fabian Zink, Ulrich Wachter, Felix Hezel, Xiaomin Zhang, Andrea Hoffmann, Michael Gröger, Clair Hartmann, Holger Gässler, Thomas Datzmann, Tamara Merz, Andreas Hellmann, Christine Kranz, Enrico Calzia, Peter Radermacher, David Alexander Christian Messerer

**Affiliations:** ^1^ Institute of Anesthesiological Pathophysiology and Process Engineering, University Hospital Ulm, Ulm, Germany; ^2^ Clinic for Anesthesia and Intensive Care, University Hospital Ulm, Ulm, Germany; ^3^ Department of Anaesthesiology, Intensive Care Medicine, Emergency Medicine and Pain Therapy, Federal Armed Forces Hospital Ulm, Ulm, Germany; ^4^ Institute of Analytical and Bioanalytical Chemistry, Ulm University, Ulm, Germany; ^5^ Department of Transfusion Medicine and Hemostaseology, Friedrich-Alexander University Erlangen-Nuremberg, University Hospital Erlangen, Erlangen, Germany

**Keywords:** hemorrhagic shock, innate immunity, mitochondrial respiration, reactive oxygen species, immunometabolism, metabolic flux analysis, metabolic modeling, hydrogen sulfide

## Abstract

**Introduction:**

Sodium thiosulfate (Na_2_S_2_O_3_), an H_2_S releasing agent, was shown to be organ-protective in experimental hemorrhage. Systemic inflammation activates immune cells, which in turn show cell type-specific metabolic plasticity with modifications of mitochondrial respiratory activity. Since H_2_S can dose-dependently stimulate or inhibit mitochondrial respiration, we investigated the effect of Na_2_S_2_O_3_ on immune cell metabolism in a blinded, randomized, controlled, long-term, porcine model of hemorrhage and resuscitation. For this purpose, we developed a Bayesian sampling-based model for ^13^C isotope metabolic flux analysis (MFA) utilizing 1,2-^13^C_2_-labeled glucose, ^13^C_6_-labeled glucose, and ^13^C_5_-labeled glutamine tracers.

**Methods:**

After 3 h of hemorrhage, anesthetized and surgically instrumented swine underwent resuscitation up to a maximum of 68 h. At 2 h of shock, animals randomly received vehicle or Na_2_S_2_O_3_ (25 mg/kg/h for 2 h, thereafter 100 mg/kg/h until 24 h after shock). At three time points (prior to shock, 24 h post shock and 64 h post shock) peripheral blood mononuclear cells (PBMCs) and granulocytes were isolated from whole blood, and cells were investigated regarding mitochondrial oxygen consumption (high resolution respirometry), reactive oxygen species production (electron spin resonance) and fluxes within the metabolic network (stable isotope-based MFA).

**Results:**

PBMCs showed significantly higher mitochondrial O_2_ uptake and lower ${\text{O}}_{2}^{•-}$ production in comparison to granulocytes. We found that in response to Na_2_S_2_O_3_ administration, PBMCs but not granulocytes had an increased mitochondrial oxygen consumption combined with a transient reduction of the citrate synthase flux and an increase of acetyl-CoA channeled into other compartments, e.g., for lipid biogenesis.

**Conclusion:**

In a porcine model of hemorrhage and resuscitation, Na_2_S_2_O_3_ administration led to increased mitochondrial oxygen consumption combined with stimulation of lipid biogenesis in PBMCs. In contrast, granulocytes remained unaffected. Granulocytes, on the other hand, remained unaffected. ${\text{O}}_{2}^{•-}$ concentration in whole blood remained constant during shock and resuscitation, indicating a sufficient anti-oxidative capacity. Overall, our MFA model seems to be is a promising approach for investigating immunometabolism; especially when combined with complementary methods.

## 1 Introduction

Treatment with hydrogen sulfide (H_2_S) resulted in controversial data in clinically relevant, long-term large animal models of hemorrhage and subsequent resuscitation, inasmuch as unchanged, attenuated and aggravated organ dysfunction were reported (1–5). These divergent findings were at least in part due the fact that depending on the dosing and timing, model and mode of administration, H_2_S exerted either anti- (6–10) or pro-inflammatory (11, 12) effects. Moreover, during hemorrhage and subsequent resuscitation, H_2_S caused variable effects on whole body energy expenditure (1, 3, 13–15), at least in part as a result of its concentration-dependent effects on mitochondrial respiration (16, 17). However, any H_2_S-related effect on energy metabolism may assume particular importance for the inflammatory response, since recent publications have reported on the involvement of H_2_S in dysregulation of the immune response by alterations in the immunometabolism, specifically suppression of glycolysis (18); while other sources reported on increased glycolysis and stimulation of lipid biosynthesis from glutamine by carboxylation of α-ketoglutarate (αKG) (19, 20).

Sodium thiosulfate (Na_2_S_2_O_3_) is a H_2_S-releasing agent (21, 22) that is recognized as an antidote in cyanide poisoning (23) as well as for its mitigation of cisplatin-induced side effects (24). Administration of Na_2_S_2_O_3_ exerted organ-protective effects in murine neuronal ischemia reperfusion injury (25, 26), LPS-induced lung injury, and polymicrobial sepsis (27). Beneficial effects of Na_2_S_2_O_3_ treatment were attributed to its anti-inflammatory, anti-oxidative and/or hypometabolic characteristics (26–28). In this context, the potential effects of Na_2_S_2_O_3_ on metabolism and mitochondrial respiration are poorly understood.

Furthermore, the cellular metabolic network has a crucial impact on inflammation and immune function: Systemic inflammation activates immune cells, which in turn show cell type-specific metabolic plasticity with modifications of mitochondrial respiratory activity. These metabolic changes were demonstrated to not only impact, but determine immune cell function (29–32). Many innate immune cells, like granulocytes, heavily rely on glycolysis and consume little oxygen. This is further enhanced upon activation, when these cells engage in glycolysis despite sufficient availability of oxygen, a phenomenon termed Warburg effect initially observed in cancer cells (32, 33). In addition to increasing the glycolytic rate, granulocytes also increase their activity through the pentose phosphate pathway (PPP), regenerating nicotinamide adenine dinucleotide phosphate (NADPH) in the process, an important cofactor for the NADPH oxidase. This enzymatic complex is a major source of the anti-microbial reactive oxygen species (ROS) superoxide ( ${\text{O}}_{2}^{•-}$ ) and, therefore, hydrogen peroxide (H_2_O_2_), which play a crucial role in innate immune defense (33, 34). In contrast, resting lymphocytes show lower rates of glycolysis and primarily rely on oxidizing glucose-derived pyruvate in the tricarboxylic acid (TCA) cycle, while generating ${\text{O}}_{2}^{•-}$ radicals as a byproduct of oxidative phosphorylation (OXPHOS) (32, 35). After activation, higher ATP demand is met by an increase in the rate of glycolysis, while fatty acid oxidation is repressed to guarantee the supply of substrates for membrane synthesis (33, 36). These mechanisms of metabolic adaptation to inflammatory stress provide a complex and highly plastic network that is distinct for each cell type (37).

Tracing individual molecules through metabolic pathways is an ongoing challenge. We have developed a model for ^13^C-based metabolic flux analysis (MFA) that utilizes glucose and glutamine tracers to be able to investigate the effect a factor might have on immunometabolism. Incubating cells with stable, non-radioactive isotope-labeled nutrients yields ^13^C labeling patterns on important metabolites which can be detected by gas chromatography/mass spectrometry (GC/MS) (38–40).

Concerning MFA, one of the most frequent strategies is to analyze labeling patterns of metabolites and deduce relative pathway utilization (39, 41). When going further and transforming these labeling patterns into fluxes, it is paramount to assess precision and accuracy. Bayesian analysis is one of the most straight forward and reliable ways to analyze error propagation and receive accurately assessed error bounds of calculated fluxes (42). We therefore propose our sampling-based model as a suitable method for estimating fluxes within the metabolic network. A preliminary version of this model has already been applied to study the effects of glucocorticoids on macrophage metabolism (43) and, in a more rudimentary form, to investigate the effect of acute subdural hematoma-induced brain injury on peripheral blood mononuclear cell (PBMC) metabolism (44).

To the best of our knowledge, data is scarce for metabolic flux during hemorrhagic shock in general and especially for interventions potentially affecting metabolism and mitochondria, such as the administration of Na_2_S_2_O_3_ and/or H_2_S. Therefore, we applied our novel MFA method to isolated circulating immune cells and tested whether Na_2_S_2_O_3_ has an impact on the metabolism of innate immunity during experimental hemorrhage and subsequent resuscitation.

## 2 Materials and methods

### 2.1 Animal procedures

The reported data utilized material obtained during a recently published study investigating the effect of Na_2_S_2_O_3_ in a long-term porcine model of hemorrhage (5). Experiments were conducted according to the National Institutes of Health Guidelines on the Use of Laboratory Animals and the European Union ‘Directive 2010/63 EU on the protection of animals used for scientific purposes’ after approval by the University of Ulm Animal Care Committee and the Federal Authorities for Animal Research (Regierungspräsidium Tübingen, Germany, Reg.-Nr. 1341, date of approval 02.05.2017). A total of 17 adult Bretoncelles-Meishan-Willebrand (BMW) pigs of both sexes (7 castrated males, 10 females) with a median weight of 62 kg (interquartile range (IQR) 56;67) and a median age of 15 months (IQR 13;16) were included in this study. The STS group included 4/5 male-castrated/female pigs and the vehicle control group consisted of 3/5 male-castrated/female animals. The BMW strain is characterized by a decreased activity of the von Willebrand factor resulting in a coagulatory state similar to that of human blood (45, 46).

### 2.2 Anesthesia and surgery

Anesthesia and surgical instrumentation are described in detail in Messerer et al.; analyzing the impact of Na_2_S_2_O_3_ on organ damage within the same cohort (5). Briefly, animals were randomly assigned to a control (n = 8) or Na_2_S_2_O_3_-treatment group (n = 9), and the staff performing the experiment was blinded to the group assignment. Hemorrhagic shock was induced by passive removal of the blood with a titration of mean arterial blood pressure (MAP) to 40 ± 5 mmHg. After 3 h of hemorrhage, swine underwent resuscitation up to a maximum of 68 h, while noradrenaline was continuously administered intravenously to restore the MAP to pre-shock levels. At 2 h of shock, animals received either vehicle (NaCl 0.9%) or Na_2_S_2_O_3_ (25 mg/kg/h for 2 h, thereafter 100 mg/kg/h until 24 h after shock, Dr. Franz Köhler Chemie GmbH, Bensheim, Germany) according to their group assignment. The choice of dose and time of administration is explained in detail in Messerer et al. (47). Briefly, it was performed in accordance with previous studies as well as established concentrations for treatment of cyanide poisoning (23, 47, 48).

### 2.3 Cell isolation from whole blood

**Figure 1:**
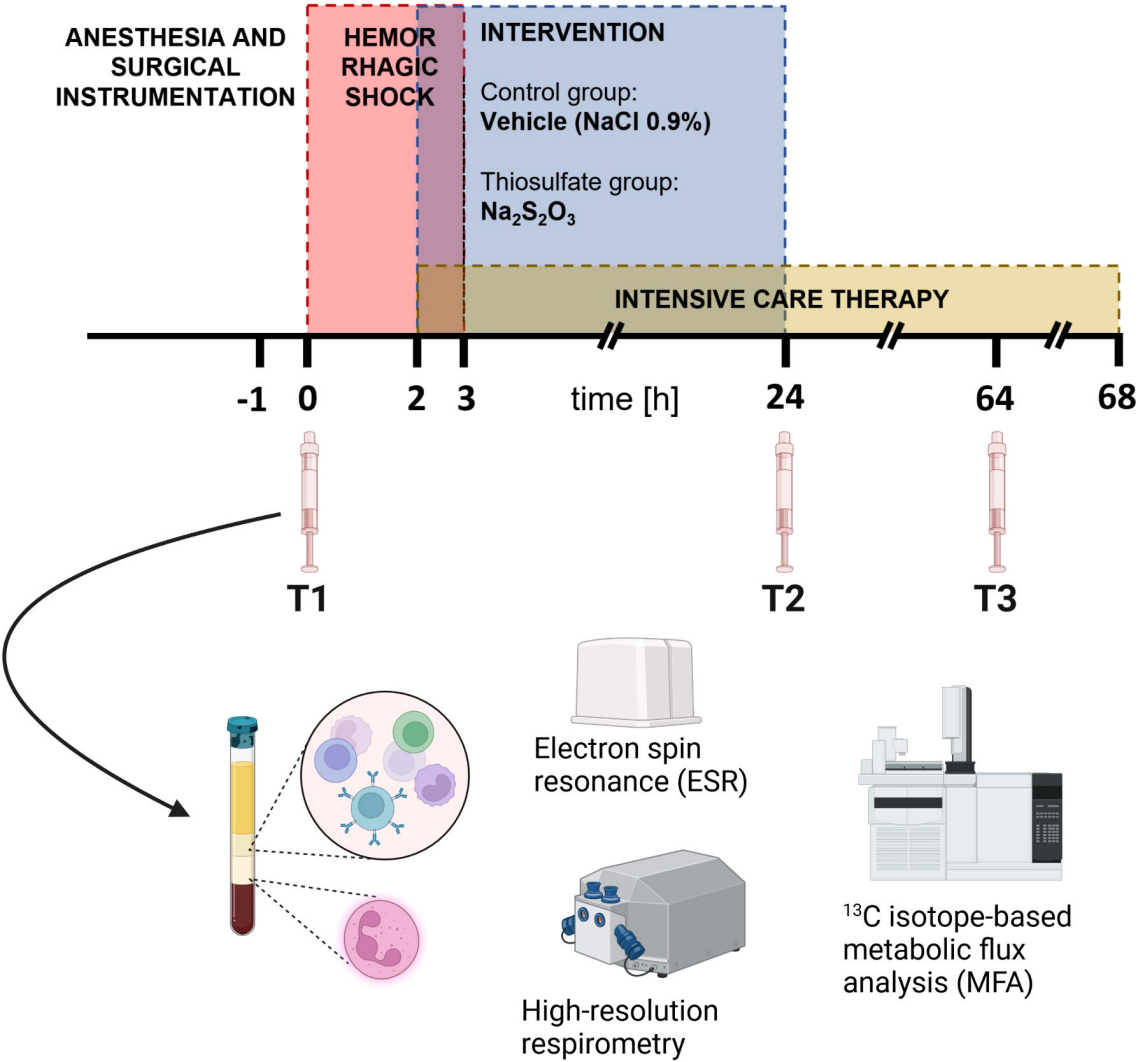
Experimental setup. In the red period, pigs were subjected to hemorrhagic shock. After 3 h of shock, animals underwent resuscitation up to a maximum of 68 h. In accordance with their group assignment, they received either Na_2_S_2_O_3_ (n = 9, 25 mg/kg/h for 2 h, thereafter 100 mg/kg/h until 24 h after shock) or vehicle (n = 8, NaCl 0.9%) during the blue-marked period. The Figure is adapted from (5).

Blood was drawn after the induction of anesthesia and a subsequent stabilization period (T1) as well as 24 h (T2) and 64 h (T3) after shock induction (**Figure 1**). 14 lithium-heparin (LiHep) monovettes with a volume of 9 mL each (Sarstedt, Nümbrecht, Germany) were used to collect approximately 120 mL of arterial whole blood each time point. After 1:1 dilution with PBS (without CaCl_2_, MgCl_2_), the drawn blood was layered onto a two density gradient solutions (9 mL 1.119 and 8 mL 1.088 g/mL solution, Pancoll, PAN Biotech, Aidenbach, Germany). Centrifugation at 764 g without break at room temperature (RT) for 20 min resulted in a PBMC top layer and a bottom layer containing red blood cells (RBCs) and granulocytes. The cells were resuspended in water for short-time osmotic lysis to remove residual RBCs before stopping the reaction with 10 × PBS to avoid lysis of leukocytes. Osmotic lysis was applied to PBMCs only once, while three procedures were required for granulocytes due to the high fraction of RBCs in the bottom layer. After removal of all RBC contamination, cells were washed once with 1 × PBS and subsequently counted in a Neubauer counting chamber. On average, isolated cells could be used for experiments experiments 2 – 3 h after blood withdrawal was firstly initiated.

### 2.4 High resolution respirometry

Mitochondrial respiration was measured by high-resolution respirometry using the Oroboros^®^ Oxygraph-2K (Oroboros Instruments, Innsbruck, Austria). This device allows for simultaneous recording of the O_2_ concentration in two parallel chambers calibrated for 2 mL of mitochondrial respiration medium MiR05 (49). This medium contains 110 mM D-sucrose (Sigma Aldrich, St. Louis, MO, USA), 60 mM K-lactobionate (Sigma Aldrich, St. Louis, MO, USA), 0.5 mM ethylene glycol tetra acetic acid (Sigma Aldrich, St. Louis, MO, USA), 1 g/L bovine serum albumin free from essential fatty acids (Sigma Aldrich, St. Louis, MO, USA), 3 mM MgCl_2_ (Scharlau, Hamburg, Germany), 20 mM taurine (Sigma Aldrich, St. Louis, MO, USA), 10 mM KH_2_PO_4_ (Merck, Darmstadt, Germany), 20 mM HEPES (Sigma Aldrich, St. Louis, MO, USA), adjusted to pH 7.1 with KOH and equilibrated with 21% O_2_ at 37°C. Directly after cell isolation, 10 × 10^6^ PBMCs/granulocytes suspended in MiR05 were filled into a chamber and stirred at 750 rpm. Sealing the chambers of the device according to the manufacturers protocol started the continuous recording of mitochondrial respiration. Quantification of the oxygen flux (*J*O_2_) was based on the rate of change in the O_2_ concentration in the chambers and normalized for the cell number. Once the chambers were sealed, specific analysis of mitochondrial respiratory function was achieved by sequential injections of substrates and inhibitors into the respiration medium. Firstly, routine respiration was recorded once a stable *J*O_2_-value was achieved after closing the chambers. Subsequently, 2.5 μM oligomycin was injected to block the ATP-synthase. This yielded the LEAK-state, which represents the respiratory activity required to maintain a stable membrane potential in absence of ATP-turnover. The titration of carbonyl cyanide p-(trifluoromethoxy)-phenylhydrazone (FCCP) in 1 µM steps allowed to achieve the maximum respiratory activity in the uncoupled state (ETS-state). The ETS state corresponds to state 3 as defined in Chance and Williams et al. (50) and is neither limited by substrate availability, cell energy demand, nucleotide availability or ATP synthase activity. Finally, 0.5 μM rotenone + 5 μM antimycin were added to block complex I and III respectively, yielding the residual (non-mitochondrial) oxygen consumption.

### 2.5 Quantification of reactive oxygen species

${\text{O}}_{2}^{•-}$ concentration in whole blood was determined immediately after blood removal. 25 µL of whole blood were mixed with an aliquot of 25 µL freshly thawed CMH (1-Hydroxy-3-methoxycarbonyl-2,2,5,5-tetramethylpyrrolidine) spin probe solution. The CMH solution contained 400 µM CMH spin probe, 25 µM deferoxamine, and 5 µM diethyldithiocarbamate to chelate transition metal ions in Krebs-HEPES-Buffer (KHB) (Noxygen, Elzach, Germany). After mixing whole blood with CMH spin probe solution, it was transferred to a 50 µL glass capillary, sealed, and measured with an EMXnano electron spin resonance (ESR) spectrometer (Bruker, Billerica, MA, USA) after 5 min incubation at 37°C (Bio-III, Noxygen, Elzach, Germany). The device settings are detailed in the Supplements. Radical concentration was quantified by comparison with a series of CP° (3-Carboxy-2,2,5,5-tetramethyl-1-pyrrolidinyloxy) radical standards solved in KHB. As a blank sample, KHB added to the respective amount of CMH spin probe solution was measured and subtracted from the sample value.

For determination of radical production by immune cells, 25 µL of a cell suspension containing 2.5 × 10^6^ cells/mL RPMI 1640 medium (Glucose 1.8 mg/mL, Glutamine 0.6 mg/mL, NaHCO_3_ 100 µg/mL) were mixed with 25 µL of CMH spin probe solution. In contrast to whole blood, cell samples were measured over a 30 min interval to calculate the radical production rate. A sample of RPMI 1640 medium mixed 1:1 with CMH spin probe solution was used as a blank value for measuring cell suspensions and subtracted from sample values. Data were evaluated with the Xenon_nano software (version 1.3; Bruker BioSpin GmbH, Rheinstetten, Germany) and Microsoft Excel. Results regarding ROS determination by ESR were included in a dissertation by one of our co-authors (51). Additionally, the extracellular H_2_O_2_ concentration was determined in a suspension of 1 × 10^6^ PBMCs/granulocytes in 100 µL PBS after 30 min at RT. A three-electrode setup that has been previously thoroughly described was used for this purpose (52). The determination of the H_2_O_2_ concentration was not performed for each animal due to limited availability of the measurement device.

### 2.6 Stable isotope incubation and detection of metabolites

For investigating nutrient utilization of cells, we incubated three times 5 × 10^6^ freshly isolated cells in parallel in 1 mL RPMI containing one of the following tracers: 1,2-^13^C_2_-labeled glucose, ^13^C_6_-labeled glucose, and ^13^C_5_-labeled glutamine (Cambridge Isotope Laboratories, Andover, MA, USA). Concentrations are specified in the **Table S3** in the Supplements. The pH of the medium was adjusted to 7.4 before experimentation through addition of 1M HCl or NaOH. After incubation at 37°C for 2 h, cells were spun down and 850 µL of the supernatant was transferred to a crimp neck glass vial. The vial was frozen upside down at −20°C for later GC/MS analysis of ^13^CO_2_ production and lactate released into the medium. The cell pellet was washed once with PBS and subsequently stored at −80°C after removal of all liquid. For MFA analysis, we required both the supernatant (^13^CO_2_ production; mass isotopomer distributions (MIDs) of secreted lactate) and the cell pellet (MIDs of the selected metabolites lactate, glutamate, and aspartate). Samples were stored for 1 – 2 months until analysis.

The cumulative cellular ^13^CO_2_ production was estimated by enrichment analysis from the spiked amount of CO_2_ released from NaHCO_3_ in the supernatant (**Table S1**). The frozen supernatant was thawed, and 25 µL 1 M HCl were injected through the septum into the liquid to drive out CO_2_ into the gaseous phase. For each vial, 10 replicates of 5 µL headspace gas each were injected into the GC/MS system (Agilent 6890 GC/5975B MSD, Agilent Technologies, Waldbronn, Germany) while analyzing the m/z of 44 and 45; corresponding to unlabeled and labeled CO_2_, respectively. The average ratio of ^13^CO_2_/^12^CO_2_ amounted to 0.56% with an average standard error of 2% of the nominal value.

After ^13^CO_2_ detection from the supernatant, we analyzed lactate secretion into the medium. Determination was performed by taking two aliquots of 100 µL supernatant and adding 500 µL acetonitrile. Samples were centrifuged at RT (13000 rpm for 5 min) and afterwards decanted into vials suited for derivatization. After drying in a Savant2010 SPD 2010 SpeedVac concentrator (Thermo Scientific, Waltham, MA, USA) (45°C, 14 mTorr) for about 50 min, derivatization was initiated with 100 µL acetonitrile and 25 µL N-(tert-butyldimethylsilyl)-N-methyltrifluoroacetamide (MTBSTFA). Samples were incubated at 80°C for 1 h with the lid closed and afterwards transferred into GC/MS vials. One of the initial 100 µL samples was incubated with 1 µg of internal standard (IS, corresponding to 20 µL of 50 µg/mL ^13^C_3_ sodiumlactate solution) for 10 min beforehand to serve for quantification. A variety of essential calibration samples were prepared in replicates: 0.1 µg/0.2 µg/0.5 µg/0.75 µg/1 µg of lactate with an additional 1 µg of IS each, 1 µg of IS only, blank RPMI, and RPMI with 1 µg of IS. Details of calibration and quantification are specified in the Supplements.

For metabolite extraction from the cell pellet, 100 µL cold H_2_O was added to the frozen pellets. The mixture was vortexed and sonicated for 10 min. Subsequently, 500 µl acetonitrile was added and the samples were centrifuged for 5 min at 13000 rpm. All samples were decanted into vials and dried for derivatization. Steps of derivatization follow those of lactate determined from medium. Standard mixes with 0.1 µg/0.2 µg/0.5 µg/0.75 µg/1 µg of analytes were prepared as control: the first with the respective amounts of lactate; the second with aspartate, glutamine, and glutamate.

For GC/MS detection, we used selected ion monitoring for optimal signal to noise ratios. Details of device settings and m/z of measured TBDMS derivatives are specified in the Supplements. Peak area integration was performed with our in-house program and MIDs were converted into carbon mass distributions (CMD) with a correction matrix approach (53, 54). MIDs were corrected for all isotopic interferences except for the natural ^13^C abundance, which is included in our CMDs.

### 2.7 Metabolic flux analysis

For MFA, we established a combined model for glycolysis, the PPP and TCA cycle, which has been previously described and utilized in Stifel et al. (43). It predicts ^13^C mass distributions on metabolites based on flow rates of the metabolic system by utilizing the EMU concept (40, 55–57) and was implemented in RStan [R interface to Stan, a tool for Bayesian analysis (58)]. Comparing predictions for ^13^C mass distributions with the corresponding GC/MS measurements (section 2.6) using sampling-based Bayesian statistics allowed for identifying suitable fluxes within the network. It further estimated how the precision in measurements affects the precision of estimated fluxes, including standard deviations and confidence intervals. Conveniently, unidentifiable fluxes can be recognized by wide confidence ranges.

Our PPP estimation is built on the same method as the one used by Lee, Katz, and Rognstad (59, 60) that is based on the assumption that PPP utilization can be represented as a shift in the label (‘carbon scrambling’) of the top carbon atoms of PPP metabolites. For this approach, usually only the m+1/m+2 ratio on lactate would be used as a proxy for triose labeling using a 1,2-^13^C_2_-labeled glucose input, but we expanded the method so that the complete CMD of the full metabolite as well as the CMD of the lactate fragment across carbon 2 and 3 were taken into account. The model firstly estimated relative fluxes from GC/MS measurements and subsequently utilized ^13^CO_2_ production and the secretion of lactate into the medium to transform these relative fluxes into absolute values. The parallel tracer setup of 1,2-^13^C_2_-labeled glucose, ^13^C_6_-labeled glucose, and ^13^C_5_-labeled glutamine enabled improved flux determination, as the estimated fluxes must apply to sets of measurements obtained from each tracer. The details of the metabolic model are available in the Supplements.

### 2.8 Statistical analysis

17 BMW pigs were included in this study. Animals were randomly assigned to a control vehicle (n = 8) or Na_2_S_2_O_3_-treatment group (n = 9). Data are presented as median with IQR and the number of animals that could be included in the corresponding analyses are indicated in the respective figure legends. Missing values at later time points indicate animals with premature experiment termination in accordance with a list of predetermined criteria (5). Statistical and graphical presentation was performed with GraphPad Prism 9, version 9.4.1 (GraphPad Software Inc., La Jolla, CA, USA). Experimental data was considered to be non-parametric due to small sample sizes. We conducted the comparison between groups with Mann-Whitney U tests, while the effect of time within one group was analyzed with the Kruskal-Wallis rank sum test and a *post hoc* Dunn’s multiple comparisons test. **Figure 1** uses templates provided by www.biorender.com.

## 3 Results

In general, the administration of Na_2_S_2_O_3_ neither altered survival nor dramatically impacted cardiocirculatory parameters, biomarkers of organ damage, or inflammatory markers; as previously published in detail in Messerer et al. (5).

### 3.1 Na_2_S_2_O_3_ administration significantly increased mitochondrial oxygen consumption in PBMCs but not in granulocytes

**Figure 2:**
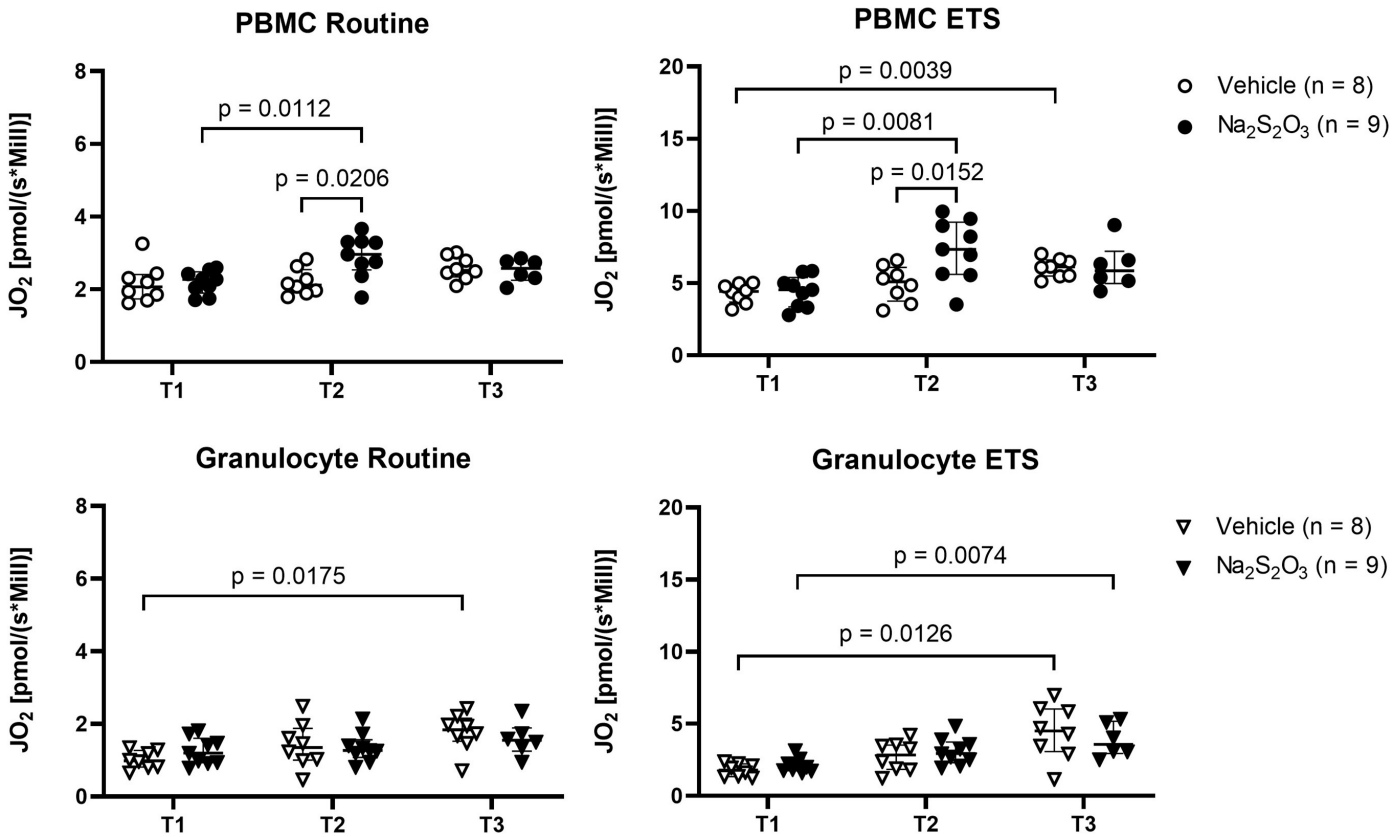
Mitochondrial oxygen consumption of PBMCs and granulocytes. *J*O_2_ was measured as baseline (routine) respiration and ETS with high resolution respirometry at indicated time points at the time points T1 (before shock), T2 (24 h post shock) and T3 (64 h post shock). Individual data of vehicle-treated animals are indicated as bright symbols; Na_2_S_2_O_3_-treated animals as dark ones. PBMCs are displayed as circles and granulocytes as triangles. Significant p values are presented in the graphs; Mann-Whitney U test for intergroup differences, Kruskal-Wallis rank sum test for time-related effects. Data are presented as median with IQR.

To determine mitochondrial oxygen consumption, we evaluated routine respiration and ETS capacity; the former describing baseline respiration and the latter representing the maximum capacity of the mitochondria independent of factors like substrate availability, oxygen availability, ATP synthase activity and cell energy demand (50). Since the respective treatment was initiated at 2 h of shock, variances in groups caused by differential treatment should become apparent at the T2 (24 h after shock) or T3 (64 h after shock) time points, while baseline T1 was expected to be comparable (**Figure 1**). Both routine and ETS mitochondrial oxygen consumption increased over the course of the experiment for PBMCs and granulocytes (**Figure 2**). Generally, PBMCs demonstrated higher respiration than granulocytes. Interestingly, Na_2_S_2_O_3_-treated PBMCs showed a significantly higher oxygen consumption compared to the vehicle-treated cells at T2, which was not present in granulocytes. By T3, this intergroup difference disappeared.

### 3.2 Whole blood radicals and immune cell ROS production did not significantly differ between groups

**Figure 3:**
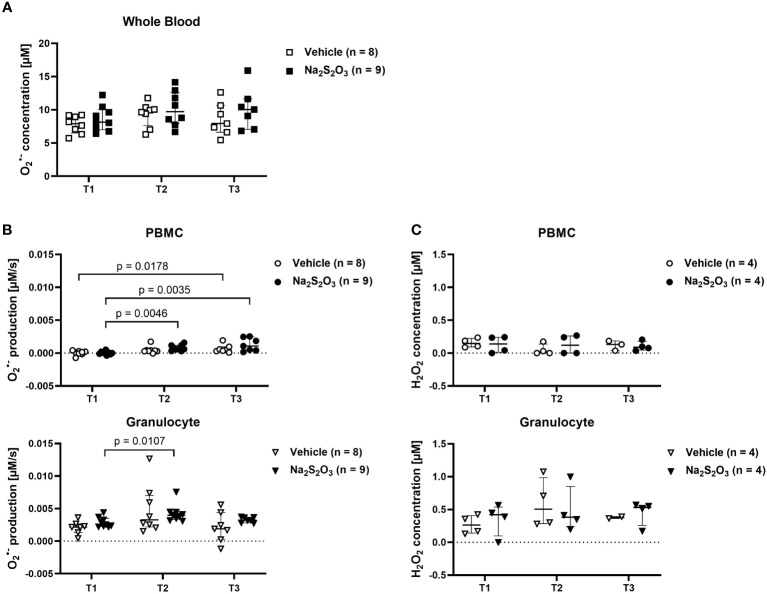
Radical production in PBMCs and granulocytes. ROS measured by ESR or electrochemical detection at the indicated time points at the time points T1 (before shock), T2 (24 h post shock) and T3 (64 h post shock). Vehicle-treated animals are indicated as bright symbols; Na_2_S_2_O_3_-treated animals as dark ones. PBMCs are displayed as circles and granulocytes as triangles. **(A)** ${\text{O}}_{2}^{•-}$ radical concentration in whole blood quantified by ESR. **(B)** ${\text{O}}_{2}^{•-}$ radical production in PBMCs and granulocytes quantified by ESR. **(C)** H_2_O_2_ concentration in PBMCs and granulocytes quantified by electrochemical detection after incubation at RT for 30 min. Significant p values are presented in the graphs; Mann-Whitney U test for intergroup differences, Kruskal-Wallis rank sum test for time-related effects. Data are presented as median with IQR.

The capability to generate ROS was uncompromised, as determined by ESR spectroscopy, the gold standard for radical measurements. As depicted in **Figure 3**, differences in production of ${\text{O}}_{2}^{•-}$ of both cell types as well as ${\text{O}}_{2}^{•-}$ levels in whole blood were non-significant between both groups at all times of measurement. When it comes to Regarding cell type specific effects, granulocytes demonstrated higher superoxide production levels than PBMCs. PBMCs slightly increased ${\text{O}}_{2}^{•-}$ production over the course of the experiment, while granulocytes displayed significantly higher production levels at T2, which returned to baseline at the end of the experiment (**Figure 3B**). To further confirm the findings obtained by ESR, we determined H_2_O_2_ produced by PBMCs or granulocytes electrochemically and found it mirroring the ESR-measured pattern despite utilizing completely different means of detection and measuring different oxidative agents (**Figure 3C**). However, only few samples could be analyzed for their H_2_O_2_ concentration, making it difficult to obtain reliable statistical informative values.

### 3.3 Na_2_S_2_O_3_ administration led to a cell type-specific transient reduction of the flow mediated by the citrate synthase flux with an interlinked increase in mitochondrial oxygen consumption

**Figure 4:**
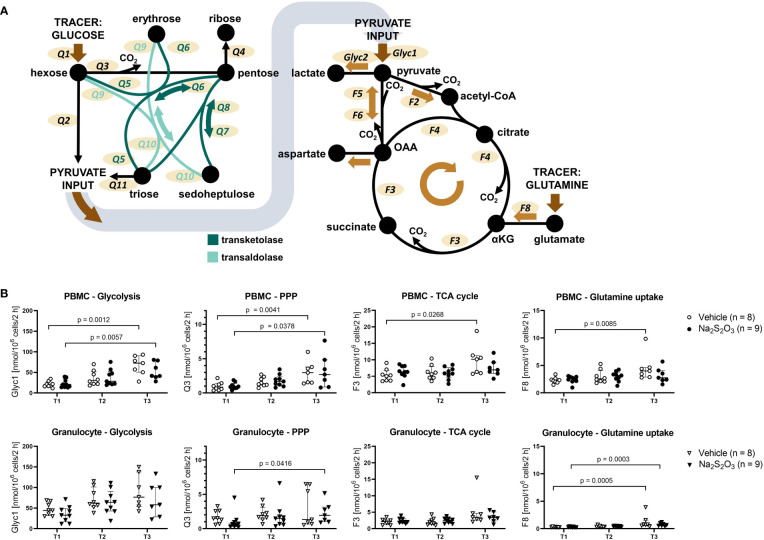
Metabolic fluxes of porcine immune cells calculated from ^13^C labeling patterns on metabolites. **(A)** Visualization of the model used for Bayesian sampling. Left: PPP model. Right: TCA cycle model. Glyc: glycolytic flux. Q: flux within the PPP. F: flux within the TCA cycle. **(B)** Glycolytic, PPP and TCA cycle fluxes of PBMCs and granulocytes. Animals were treated either with vehicle (n = 8, bright symbols) or Na_2_S_2_O_3_ (n = 9, dark symbols). αKG: α-ketoglutarate, OAA: oxaloacetate. Significant p values are indicated in the graphs; Mann-Whitney U test for intergroup differences, Kruskal-Wallis rank sum test for time-related effects. Data are presented as median with IQR.

We analyzed immune cell metabolism with a model formulated in RStan (visualized in **Figure 4A**). Our ^13^C-based MFA method managed to successfully reproduce textbook knowledge regarding cell type specific preferences in metabolism such as granulocytes displaying higher glycolytic rates while PBMCs rather utilized the TCA cycle (**Figure 4B**) (32, 61, 62). Moreover, PBMCs displayed much higher glutamate metabolism (F8) in comparison to granulocytes: about 40 – 50% (Median 0.44; IQR 0.39; 0.48) of the main TCA cycle flux F3 originated from glutamine *via* glutamate in PBMCs, while it only added up to 13% for granulocytes (IQR 0.11; 0.19).

Although the glutamate input also slightly increased with shock progression for granulocytes, it only reached 22% at its highest at T3. PPP utilization was similar for both cell subsets, however, granulocytes showed completely divergent PPP utilization at the last measurement time point. So far, we could not find correlations with other parameters that could explain the two extremes.

**Figure 5:**
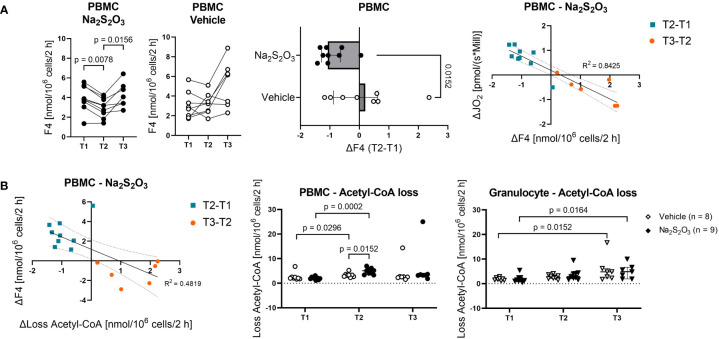
The effect of Na_2_S_2_O_3_ on porcine PBMCs. Metabolic fluxes calculated from ^13^C labeling patterns on metabolites. PBMCs are displayed as circles and granulocytes as triangles. Animals were treated either with vehicle (n = 8, bright symbols) or Na_2_S_2_O_3_ (n = 9, dark symbols). **(A)** F4 trends in PBMCs in vehicle vs Na_2_S_2_O_3_-treated animals and correlations between the difference in F4 and the difference in routine respiration over time. **(B)** Acetyl-CoA loss of PBMCs and granulocytes. Correlation between the difference in F4 and the difference in Acetyl-CoA loss over time. Significant P values are indicated in the graphs; Mann-Whitney U test for intergroup differences, Kruskal-Wallis rank sum test for time-related effects. Data are presented as median with IQR. Correlations are presented with 95% confidence intervals.

Generally, cell metabolism reacted similarly in response to the intensive care treatment, inasmuch almost all fluxes gradually increased from T1 to T3 (**Figure 4B**). These changes were significant for PMBCs, while granulocytes only demonstrated a slight trend. The only notable exception were TCA cycle fluxes, which remained mostly constant, and PBMCs from Na_2_S_2_O_3_-treated animals, where citrate synthesis significantly decreased at T2 (**Figure 5A**).

When it comes to the effect of Na_2_S_2_O_3_ on immunometabolism, it seems like neither glycolytic nor PPP or TCA cycle fluxes of granulocytes were impacted by its administration (**Figure 4B**). While PBMCs also failed to show intergroup differences regarding glycolytic and PPP metabolism, there was a notable and significant difference in F4, the flux from oxaloacetate (OAA) over citrate to α-ketoglutarate, pertaining to the change over the course of the experiment (**Figure 5A**): Na_2_S_2_O_3_-treated PBMCs significantly decreased their F4 flux from T1 to T2, while this was not the case for vehicle-treated PBMCs. Intriguingly, this difference in F4 between measurement time points highly correlated with the intergroup differences observed in routine respiration (R^2^ = 0.8425, p < 0.0001). This negative linear regression was neither present in the control group nor in granulocytes. PBMCs from Na_2_S_2_O_3_-treated animals further displayed a significantly increased loss in acetyl-CoA, representing the amount of acetyl-CoA exiting the described subsystems of **Figure 4A**, compared to vehicle-treated ones at T2 (**Figure 5B**). As an indicator of potential reductive carboxylation, we compared the m+3 ^13^C label on the complete aspartate fragment between vehicle and Na_2_S_2_O_3_-treated animals at T2. There was no difference between groups (Vehicle: 1.94%, Na_2_S_2_O_3_: 1.78%; p = 0.2925), implying that the higher amount of acetyl-CoA loss was not coupled to reductive carboxylation. The change in acetyl-CoA loss, like the change in mitochondrial oxygen consumption, correlated with the change in F4 (**Figure 5B**).

## 4 Discussion

We investigated the effect of Na_2_S_2_O_3_ on the metabolism of circulating immune cells in a porcine model of hemorrhage and resuscitation over a period of max 68 h. The main findings included that Na_2_S_2_O_3_ administration for a 24 h period i) did not impact granulocyte metabolism or ROS production, while ii) it increased the mitochondrial oxygen consumption of PBMCs at the end of the administration period. Furthermore, iii) in PBMCs there was a transient reduction of the citrate synthase flux, which significantly correlated with the increase in mitochondrial oxygen consumption and acetyl-CoA exiting the TCA cycle network, potentially to be utilized in lipid biogenesis.

Na_2_S_2_O_3_ administration had displayed organ-protective and anti-inflammatory effects in previous studies. Through inhibition of caspase 3, Na_2_S_2_O_3_ administration initiated anti-apoptotic effects by attenuation of cerebral ischemia (25, 63) and reduction of myocardial ischemia reperfusion injury in rats (26). The latter study also demonstrated mitochondrial preservation through Na_2_S_2_O_3_ by opening ATP-sensitive potassium channels (KATP) channels as well as preserving activity of various mitochondrial enzymes, proteins and functions involved in ROS homeostasis (26). The most prominent were NADH dehydrogenase, the major contributor to electron transport chain complex I activity and one of the main producer of the ${\text{O}}_{2}^{•-}$ radical, the malate aspartate shuttle which provides NADH for complex I, as well as peroxisome proliferator-activated receptor γ coactivator 1α (PGC-1α), which is an important anti-oxidant factor in ROS detoxification (26, 64).

As Na_2_S_2_O_3_ is an approved drug and is considered for potential intervention during shock, we investigated its impact on the immune response. When applying our novel MFA model, we reproduced known cell-specific preferences of metabolic pathways: The overall lower mitochondrial oxygen consumption (routine and ETS respiration) of granulocytes in comparison to PBMCs maps with their characteristic preference of glycolytic pathways which only require baseline TCA cycle activity and minimal glutamine uptake (32, 37). These findings were confirmed with MFA, where we found that the flux patterns follow this exact pattern. Interestingly, there was no significant difference in PPP utilization between granulocytes and PBMCs. However, it should be noted that due to us not detecting metabolites directly involved in the PPP, the margin of error for these fluxes is much higher as estimations are only based on ^13^C isotope patterns on lactate.

In terms of metabolic changes in response to shock and resuscitation, mitochondrial oxygen consumption significantly increased in both PBMCs and granulocytes, potentially due to immune activation and higher energy expenditure (32). Interestingly, whole blood ROS concentration reacted neither to Na_2_S_2_O_3_ administration, shock initiation, retransfusion, nor noradrenalin administration, which stands in contrast to literature observing an increase in oxidative activity; mainly due to ischemia reperfusion injury (65, 66). This might indicate that the anti-oxidative capacity of whole blood is too high to reflect small scale fluctuations in radical concentration and that the increase in radical concentration is mainly localized at the site of ischemic tissue.

In our experiments, Na_2_S_2_O_3_ administration impacted neither whole blood radical concentration nor immune cell superoxide production. Intriguingly, there was also no effect on glycolysis, thus posing a contrast to current findings of other groups (18, 19). These divergent findings might be due to us utilizing a porcine ex-vivo model, as Rahman et al. (18) worked with cystathionine-γ-lyase (CSE)^–/–^ mice and Carballal et al. (19) *in vitro*. However, we did observe an effect pertaining to PBMC metabolism at the end of the administration period: Cells obtained from Na_2_S_2_O_3_-treated animals had increased oxygen consumption as well a transient reduction of the citrate synthase flux. This raises the question about the impact of Na_2_S_2_O_3_ on mitochondria. H_2_S, which can be released by Na_2_S_2_O_3_ especially under hypoxic conditions, characteristically demonstrates concentration-dependent effects (17). Originally, H_2_S was mainly known for its cytotoxic properties by inhibition of cytochrome c oxidase (complex IV) (67), resulting in suppressed mitochondrial electron transport. However, after being recognized as the third gasotransmitter, a variety of beneficial effects have been reported (16, 17). At low concentrations, H_2_S stimulates mitochondria by acting as an electron donor with an effect comparable to that of NADH or FADH_2_ (17, 68, 69). Consequently, H_2_S can contribute to energetic homeostasis under conditions of impaired oxygen supply and attenuate damage due to hypoxia. Our results of increased oxygen consumption seem to support this notion. However, it is important to keep in mind that an increased oxygen consumption does not equal increased mitochondrial activity but could simply indicate a change in ATP/O_2_ ratio, e.g., less ‘effective’ ATP generation. Furthermore, it has been reported that H_2_S is mainly metabolized in a process of mitochondrial detoxification (70).

The Na_2_S_2_O_3_-induced transient reduction in the citrate synthase flux observed over the course of the experiment in the citrate synthase flux observed in PBMCs correlated with the increase correlated with the increase in both the oxygen consumption and the acetyl-CoA exiting the TCA cycle network. These findings support the notion that Na_2_S_2_O_3_ administration might contribute to lipogenesis in some circulating immune cell subsets. H_2_S-stimulated lipid synthesis from glutamine is a well-documented effect in cell culture and algae, which is characterized by acetyl-CoA generation through citrate (19, 71). To investigate whether the acetyl-CoA lost for lipid biogenesis or similar mechanisms has a glucose or a glutamine origin, we compared the ^13^C_5_-glutamine tracer-induced m+3 label on aspartate, which Zhang et al. (72) and Alkan et al. (73) reported to be indicative of reductive glutamine catabolism. While our experiment produced a significantly higher label in PBMCs than in granulocytes, there was no difference between cells originating from vehicle and Na_2_S_2_O_3_-treated animals, which speaks against the putative increase in lipogenesis being an effect induced by reductive carboxylation and rather suggests a glucose origin.

Since we utilized Na_2_S_2_O_3_ as an intervention, the question arises whether Na_2_S_2_O_3_ itself or Na_2_S_2_O_3_-released H_2_S is the acting agent in the alteration of immunometabolism. As we focused on the effect on circulating immune cells and the blood pO_2_ was always maintained within normoxemic levels, it is likely that only minor amounts of H_2_S were released. Overall, however, the dose and route of administration of Na_2_S_2_O_3_ used in this study seemed to be appropriate for maintaining immune cell integrity, as the increase in mitochondrial oxygen consumption mirrored stimulating effects observed in other groups (16, 17, 19), and no further deleterious effect could be detected.

### 4.1 Limitations of the study

As we used density gradient centrifugation to isolate immune cells, it became increasingly difficult to obtain pure PBMC populations at later time points. Low density granulocytes are a population of granulocytes found in the PBMC layer of density gradients and tend to increase in number upon inflammation and, consequently, shock and resuscitation (74, 75). As they are metabolically not particularly well defined, this fraction within the PBMC population could potentially distort results at the later time points of blood withdrawal. Moreover, as PBMCs are not a metabolically uniform population, changes in one subpopulation might be masked by others. With our MFA routine, only conclusions about the whole population but not of their subsets can be drawn. Another factor to consider is potential immune cell activation during cell purification.

### 4.2 Conclusion

In summary, our novel MFA model is a suitable tool for detecting small scale metabolic alterations in immune cells. Further studies with more homogenic populations could elucidate which PBMC subset precisely is responsible for the treatment-induced metabolic effect.

## Data availability statement

The raw data supporting the conclusions of this article will be made available by the authors, without undue reservation.

## Ethics statement

The animal study was reviewed and approved by Federal Authorities for Animal Research, Regierungspräsidium Tübingen, Germany, Reg.-Nr. 1341, date of approval 02.05.2017.

## Author contributions

Study design/planning: PR, DM, EC, CK, CH, JV, UW, TM. Data acquisition: E-MW, FZ, FH, XZ, MH, DM, AHo, MG, UW, JV, HG, AHe, CK, EC, PR, TD. Data analysis: E-MW, FZ, XZ, MH, JV, DM. Writing paper: E-MW, PR, DM. Revising paper: all authors.
