# Supplementary material for: The effect of sodium thiosulfate on immune cell metabolism during porcine hemorrhage and resuscitation
Source: Front Immunol. 2023 Feb 23;14:1125594. doi: 10.3389/fimmu.2023.1125594 (PMC9996035; doi:10.3389/fimmu.2023.1125594)
Supplement: Supplementary file 1 [file DataSheet_1.pdf]

Supplementary Material

The effect of sodium thiosulfate on immune cell metabolism and function during porcine hemorrhage and resuscitation

Eva-Maria Wolfschmitt[1,*], Melanie Hogg[1], Josef Albert Vogt[1], Fabian Zink[1], Ulrich Wachter[1], Felix Hezel[1], Xiaomin Zhang[1], Andrea Hoffmann[1], Michael Gröger[1], Clair Hartmann[2], Holger Gässler[3], Thomas Datzmann[1,2], Tamara Merz[1], Andreas Hellmann[4], Christine Kranz[4], Enrico Calzia[1], Peter Radermacher[1,*], David Alexander Christian Messerer[1,2,5]

*** Correspondence:** Eva-Maria Wolfschmitt: eva-maria.wolfschmitt@uni-ulm.de

1 Metabolic modeling in RStan

Models for metabolic flux analysis (MFA) need to consider all essential metabolic processes to achieve reliable flux determination. For this reason, we established a combined model for glycolysis, the pentose phosphate pathway (PPP), and the tricarboxylic acid (TCA) cycle, which pose the most important metabolic pathways for immune cells. We used RStan (R interface to Stan) to define this model, a library for Bayesian modeling which utilizes user-defined models and data to return posterior simulations of prior defined parameters (1). Stan uses a sampling-based statistical method. Random samples of unknown parameters, such as flow rates, are drawn and used to calculate predictions, corresponding to theoretical mass distributions of certain metabolites. If the predictions result in values close to the GC/MS measurements within the bounds of the expected measurement error, the theoretical value is considered a ‘true’ value of the underlying distribution and collected in a sampling chain. Otherwise, they are disregarded. The chain of ‘true’ samples is used to calculate median, variance, and confidence intervals. We worked with mass isotopomer distributions (MIDs) and not positional labeling; meaning we only considered the amount of labeling on the whole fragment and not the exact position of the isotopic ^{13}C label. Furthermore, our definition of ^{13}C label includes the natural ^{13}C abundance, while all other natural isotopic variances were corrected for. The supplementary file ‘Supplements_CMDs.xlsx’ contains all corrected carbon mass distributions (CMDs) and $^{13}CO_2$ productions required for model calculations.

Figure S1 is a visualization of the theoretical model used for calculations, with abbreviations being assigned in Table S1 and the list below. Each node represents a metabolite with its isotope labeling and each arrow a flux contributing to the metabolite mass distribution. Table S1 further summarizes the input-output flux balance of each metabolite. The calculation of theoretical mass distribution, e.g. through condensation of two metabolites, was based on the elementary metabolite unit (EMU) approach (2–5). To optimize for speed and to ensure efficient calculation, we reduced the model to a set of essential labeling patterns (oxaloacetate, acetyl-CoA), while the labeling on other metabolites was derived from the latter (6). In total, we worked with three Stan routines; one for determining relative fluxes contributing to mass distributions of metabolites, one for estimating lactate secretion from cell to medium and one for estimating absolute fluxes from relative flux contributions.

Abbreviations and notations:

- αKG: α-ketoglutarate pool

- ACC: acetyl-CoA pool
- CMD: carbon mass distribution
- F_n: flux within the TCA cycle
- $Glyc_n$: glycolytic fluxes
- L_n: loss of metabolite due to compartmentalization
- MID: mass isotopomer distribution
- OAA: oxaloacetate pool
- Q_n: flux within the PPP
- R_n: flux ratio within the TCA cycle
- S_n: flux ratio within the PPP
- t: tracer input

1.1 Primary model: Estimation of flux ratios

1.1.1 Pentose phosphate pathway modeling

For PPP metabolites, we only considered the upper carbon atoms (i.e $c_1 - c_4$ of sedoheptulose, $c_1 - c_3$ of hexose, and $c_1 - c_2$ of pentose), since the lower three are not impacted by transaldolase or transketolase reactions and only later interact with the triose pool (7). We termed the fluxes within the PPP Q_n and the resulting ratios of fluxes S_n. For simplification, the balances were normalized over the output of the corresponding metabolite pool (Table S1). The ratios were defined as follows:

$$S_1 = \frac{Q_1}{Q_2+Q_3+Q_6+Q_{10}};\ S_5 = \frac{Q_5}{Q_2+Q_3+Q_6+Q_{10}};\ S_6 = \frac{Q_6}{Q_5+Q_{10}};\ S_7 = \frac{Q_7}{Q_6+Q_8+Q_9+Q_{11}};\ S_{10} = \frac{Q_{10}}{Q_6+Q_8+Q_9+Q_{11}};$$

$$S_2 = \frac{Q_7}{Q_8+Q_9};\ S_8 = \frac{Q_8}{Q_4+Q_5+2Q_7};\ S_3 = \frac{Q_3}{Q_4+Q_5+2Q_7};\ S_{11} = \frac{Q_{11}}{2Q_2+Q_{11}}$$

For calculation of single carbon labeling, we defined a matrix describing the relationships of the single carbons, which match the equations used by Lee et al. and Katz et al. (7, 8). The lower-case letter indicates the symbol of the corresponding metabolite as defined in Table S1 and the subscript the position of the carbon. For example, h_3 pertains to the third carbon atom of hexose, while p_{23} describes the labeling across carbons 2 and 3 of pentose. We generalized all pentoses within the PPP as they could not be differentiated in our GC/MS routine and should contain the same labeling in theory. gc_x refers to the expected labeling across the xth carbon of the glucose input while considering the 50:50 ratio of labeled to unlabeled glucose and a variable dilution of tracer. For the $^{13}C_5$-labeled glutamine tracer, potential labeling of PPP metabolites exceeding natural labeling could be excluded as we did not observe labeling on lactate for this tracer.

$$\begin{pmatrix} -1 & & & S_5 & & 1-S_1-S_5 & & & & \\ & -1 & & & S_5 & & 1-S_1-S_5 & & & \\ & & -1 & & & & & 1-S_1-S_5 & & S_5 \\ 1-S_3-2S_8 & S_3 & & -1 & & S_8 & & S_8 & & \\ & 1-S_3-2S_8 & S_3 & & -1 & & S_8 & & S_8 & \\ 1-S_2 & & & S_2 & & -1 & & & & \\ & 1-S_2 & & & S_2 & & -1 & & & \\ & & 1-S_2 & S_2 & & & & -1 & & \\ & & & & S_2 & & & & -1 & 1-S_2 \\ & & S_6 & & & & & & 1-S_6 & -1 \end{pmatrix} \otimes \begin{bmatrix} h_1 \\ h_2 \\ h_3 \\ p_1 \\ p_2 \\ s_1 \\ s_2 \\ s_3 \\ s_4 \\ e_1 \end{bmatrix} = \begin{bmatrix} -S_1\, gc_1 \\ -S_1\, gc_2 \\ -S_1\, gc_3 \\ \\ \\ \\ \\ \\ \\ \\ \end{bmatrix}$$

Calculation of labeling patterns across two or three carbon fragments followed the same pattern. However, as some distributions were produced from labels belonging to two different metabolites, we needed to consider how the reactant CMDs impacted the product CMD. A condensation reaction between two metabolites can be described with a matrix multiplication of the respective CMDs, resulting in the CMD of the condensation product. For example, $h_3 \otimes e_1$ would refer to the condensation of the third hexose carbon and the first erythrose carbon. As an example, if we were given MIDs of two fragments undergoing condensation, one 3 carbons long (represented by c), the second two carbons long (e), we could express the MID of the first reactant as a matrix with the dimensions (3, 6). The number of rows equals the number of elements in the MID of the condensation product, while the number of columns is given by the number of elements in the second reactant. The MID of e will be kept as a vector.

$$[MID_c] = \begin{pmatrix} c_{m+0} & & \\ c_{m+1} & c_{m+0} & \\ c_{m+2} & c_{m+1} & c_{m+0} \\ c_{m+3} & c_{m+2} & c_{m+1} \\ & c_{m+3} & c_{m+2} \\ & & c_{m+3} \end{pmatrix}; MID_e = \begin{pmatrix} e_{m+0} \\ e_{m+1} \\ e_{m+2} \end{pmatrix}$$

When performing a matrix multiplication with the given matrix and vector, the result will be the following vector:

$$[MID_c] * MID_e = \begin{pmatrix} c_{m+0} * e_{m+0} \\ c_{m+1} * e_{m+0} + c_{m+0} * e_{m+1} \\ c_{m+2} * e_{m+0} + c_{m+1} * e_{m+1} + c_{m+0} * e_{m+2} \\ c_{m+3} * e_{m+0} + c_{m+2} * e_{m+1} + c_{m+1} * e_{m+2} \\ c_{m+3} * e_{m+1} + c_{m+2} * e_{m+2} \\ c_{m+3} * e_{m+2} \end{pmatrix} = MID_{ce}$$

All condensation reactions were calculated accordingly and the equations for two and three carbon fragments were defined as follows:

$$\begin{pmatrix} -1 & 1 - S_1 - S_5 \\ 1 - S_2 & -1 \end{pmatrix} \begin{bmatrix} h_{23} \\ s_{23} \end{bmatrix} = \begin{bmatrix} -S_1\, gc_{23} - S_5\, p_2 \otimes e_1 \\ -S_2\, p_1 \otimes p_2 \end{bmatrix}$$

$$\begin{pmatrix} -1 & 1 - S_1 - S_5 & & S_5 \\ 1 - S_2 & -1 & & S_2 \\ & & -1 & S_2 \\ 1 - S_3 - 2S_8 & S_8 & S_8 & -1 \end{pmatrix} \begin{bmatrix} h_{12} \\ s_{12} \\ s_{34} \\ p_{12} \end{bmatrix} = \begin{bmatrix} -S_1\, gc_{12} \\ \\ -(1 - S_2)\, h_3 \otimes e_1 \\ -S_3\, h_{23} \end{bmatrix}$$

$$\begin{pmatrix} -1 & 1 - S_1 - S_5 \\ 1 - S_2 & -1 \end{pmatrix} \begin{bmatrix} h_{123} \\ s_{123} \end{bmatrix} = \begin{bmatrix} -S_1\, gc_{123} - S_5\, p_{12} \otimes e_1 \\ -S_2\, p_{12} \otimes p_1 \end{bmatrix}$$

The input of labeled metabolites t into pyruvate was calculated by taking into account the potential 'label mirroring' of the trioses (e.g., phosphoglycerate) as well as the input of other triose labels (glycerin-3-phosphate) through the PPP:

$$t_{123} = 0.5\big((1 - S_{11})h_{123} + (1 - S_{11})h_{456}\big) + S_{11}\, tr_{123}$$

$$t_{23} = 0.5\big((1 - S_{11})h_{23} + (1 - S_{11})h_{45}\big) + S_{11}\, tr_{23}$$

For sampling of PPP parameters, we had 4 measurement CMDs as determined by GC-MS (corresponding to two lactate fragments for each of the glucose tracers (1,2-$^{13}C_2$ glucose and $^{13}C_6$ glucose)). Eleven fluxes needed to be determined (Q_x). From the five PPP flux balances of hexose, sedoheptulose, pentose, erythrose, and triose (Table S1), five of these fluxes could be deduced in relation to the other six. These were termed the 'dependent fluxes' Q_1, Q_3, Q_6, Q_8 and Q_{10}. The other six fluxes made up the 'independent fluxes' Q_2, Q_4, Q_5, Q_7, Q_9, and Q_{11}. We set Q_2 = 1000 and expected Q_7 and Q_9 to have similar values, leaving the four independent fluxes Q_4, Q_5, $Q_{7/9}$ and Q_{11} to be sampled as parameters. Later, once Q_2 was deduced from the lactate accumulation in the medium, these fluxes were scaled accordingly.

$$\begin{bmatrix} Q_1 \\ Q_3 \\ Q_6 \\ Q_8 \\ Q_{10} \end{bmatrix} = \begin{pmatrix} 1 & 1 & & & & 1 \\ & 1 & & & & 3 \\ & & 1 & & & -1 \\ & & & 1 & & -1 \\ & & & & 1 & -1 \end{pmatrix} \begin{bmatrix} Q_2 \\ Q_4 \\ Q_5 \\ Q_7 \\ Q_9 \\ Q_{11} \end{bmatrix}$$

1.1.2 Tricarboxylic cycle modeling

For the glucose-derived tracer input into the TCA cycle, we took the calculated 'pyruvate input' labeling t_x of the PPP in case of the 1,2-$^{13}C_2$-labeled and $^{13}C_6$-labeled glucose tracer, with x describing the number of carbons the labeling spans over. As the PPP was neglectable for the $^{13}C_5$-labeled glutamine tracer, we calculated this input assuming natural labeling.

Accordingly, g^x was the glutamine-derived tracer input into the system. In the case of the $^{13}C_5$-labeled glutamine tracer, 100 % of glutamine was labeled, so we only took potential dilution of the ^{13}C label into account. For both of the glucose tracers, we expected natural ^{13}C distributions for g^x.

Fluxes within the TCA cycle had the abbreviation F_n and their ratios R_n. These balances were normalized over the output of the respective metabolite pool (Table S1).

$$R_1 = \frac{2Q_2 + Q_{11}}{Glyc_2 + F_2 + F_6 + L_{Pyr}}; R_2 = \frac{F_2}{F_4 + L_{ACC}};\ R_3 = \frac{F_3}{F_5 + F_4 + L_{OAA}};$$

$$R_4 = \frac{F_4}{F_3}; R_5 = \frac{F_5}{Glyc_2 +\ F_2 + F_6 + L_{Pyr}};\ R_6 = \frac{F_6}{F_5 + F_4 + L_{OAA}}$$

In the first model, only TCA cycle ratios and not fluxes were considered. One-carbon equations for our metabolites were set up similarly to the EMU approach. The model was reduced to a set of essential labeling patterns (o_x: oxaloacetate, a_x: acetyl-CoA) (5, 6, 9), while other metabolites were calculated in relation to them. n^x describes the expected natural ^{13}C labeling on an x carbon metabolite. We used n = 0.01085 for these calculations.

$$\psi_1 = R_3(1 - R_4);\ \psi_2 = 0.5\, R_3R_4;\ \psi_3 = 1 - R_3 - R_6;\ \psi_4 = 1 - R_1 - R_5$$

$$\begin{pmatrix} -1 & & R_2R_5 & \\ & -1 & & R_2R_5 \\ & 0.5\,R_3R_4 & R_6R_5-1+0.5\,R_3R_4 & \\ & 0.5\,R_3R_4 & R_6R_5+0.5\,R_3R_4 & -1 \end{pmatrix} \begin{bmatrix} a_1 \\ a_2 \\ o_2 \\ o_3 \end{bmatrix} = \begin{bmatrix} -R_1R_2\,t_2 - (1-R_2)\,n^1 \\ -R_1R_2\,t_3 - (1-R_2)\,n^1 \\ -\psi_1\,g^1 - \psi_3\,n_1 - R_1R_6\,t_2 \\ -\psi_1\,g^1 - \psi_3\,n_1 - R_1R_6\,t_3 \end{bmatrix}$$

$$o_4 = \psi_2\,(a_1 + o_3) + \psi_1\,g^1 + R_6\,c_1 + \psi_3\,n^1$$

$$o_1 = \frac{\psi_2\,(a_1 + o_3) + \psi_1\,g^1 + R_1R_6\,t_1 + \psi_3\,n^1}{1 - R_6R_5}$$

$$pyr_i = R_1\,t_i + R_5\,o_i + \psi_4\,n^1$$

c_1 is the carbon labeling on CO_2 which contributes to the labeling in OAA through F_6. As it potentially holds labeling exceeding that of n^1 due to $^{13}CO_2$ splitting off during various PPP and TCA cycle reactions (Figure S1), we considered its CMD in the case of every tracer as a parameter.

We subsequently expanded the EMUs to two and three carbons:

$$\begin{pmatrix} 1 & -R_6 \\ -R_5 & 1 \end{pmatrix} \begin{bmatrix} o_{23} \\ pyr_{23} \end{bmatrix} = \begin{bmatrix} \psi_3\,n^2 + 2\,\psi_2 o_2 \otimes a_2 + \psi_1\,g^2 \\ R_1 t_{23} + \psi_4\,n^2 \end{bmatrix}$$

$$\begin{pmatrix} 1 & -R_6 \\ -R_5 & 1 \end{pmatrix} \begin{bmatrix} o_{12} \\ pyr_{12} \end{bmatrix} = \begin{bmatrix} \psi_3\,n^2 + \psi_2\,(a_{12} + o_{23}) + \psi_1\,g^2 \\ R_1 t_{12} + \psi_4\,n^2 \end{bmatrix}$$

$$o_{34} = \psi_2\,(a_{12} + o_{23}) + \psi_1\,g^2 + R_6\,c_1 \otimes pyr_3 + \psi_3\,n^2$$

$$\begin{pmatrix} 1 & -R_6 \\ -R_5 & 1 \end{pmatrix} \begin{bmatrix} o_{1-3} \\ pyr_{1-3} \end{bmatrix} = \begin{bmatrix} \psi_3\,n^3 + \psi_2\,(a_{12} \otimes o_2 + o_{23} \otimes a_2) + \psi_1\,g^3 \\ R_1 t_{1-3} + \psi_4\,n^3 \end{bmatrix}$$

$$o_{2-4} = \psi_2\,(a_{12} \otimes o_2 + o_{23} \otimes a_2) + \psi_1\,g^3 + R_6\,c_1 \otimes pyr_{23} + \psi_3\,n^3$$

With these CMDs, we could calculate the label across the molecular ion of OAA, from which the theoretical CMD of αKG was deduced.

$$o_{1-4} = \psi_2\,a_{12} \otimes o_{23} + \psi_1\,g^4 + R_6\,c_1 \otimes pyr_{1-3} + \psi_3\,n^4$$

$$akg_1 = R_4\,o_4 + (1 - R_4)g^1$$

$$akg_{2-5} = R_4\,a_{12} \otimes o_{23} + (1 - R_4)g^4$$

$$akg_{1-5} = R_4\,a_{12} \otimes o_{2-4} + (1 - R_4)g^5$$

Finally, we could obtain the labels of the fragments measured with GC/MS, namely glutamate at the sampling site (gt), aspartate (asp), and lactate (lac). V, K, W and Z are dilution factors at the sampling site. R_7 is the ratio of labeling originating from αKG in comparison to that coming directly from glutamine at the glutamate sampling site.

$$gt_{1-5} = \mathrm{V}(R_7\,akg_{1-5} + (1 - R_7)g^{1-5}) + (1 - V)n^5$$

$$gt_{2-5} = \mathrm{V}(R_7\,akg_{2-5} + (1 - R_7)g^{2-5}) + (1 - V)n^4$$

$$asp_{1-4} = \mathrm{K}\, o_{1-4} + (1-K)n^4$$

$$asp_{2-4} = \mathrm{K}\, o_{2-4} + (1-K)n^3$$

$$asp_{12} = \mathrm{K}\, o_{12} + (1-K)n^2$$

$$lac_{1-3} = \mathrm{W}\, pyr_{1-3} + (1-W)n^3$$

$$lac_{23} = \mathrm{W}\, pyr_{23} + (1-W)n^2$$

Parameters consisted of dilution factors (V, K, W, Z) and ratios (R_1, R_2, …). Parameters of PPP and TCA cycle networks were sampled together in one model, corresponding to a total of 21 parameters. Parameters had to fit measurements obtained for all seven fragments of each tracer, resulting in a total amount of 21 CMDs for sampling purposes (Table S2). Important parameters, their transformation and their priors are listed in Table S4.

1.2 Secondary model: Estimation of $Glyc_2$ through lactate ^{13}C labeling

We measured the ^{13}C labeling on the molecular ion of lactate in the supernatant, which we used to calculate the lactate secreted by the incubated cells (corresponding to the flux $Glyc_2$ of the following passage 1.3). To account for lactate blank values brought in during sample preparation, we ran a variety of control samples, namely 0.1 µg/0.2 µg/0.5 µg/0.75 µg/1 µg of lactate with an additional 1 µg of internal standard (IS, corresponding to 20 µL of 50 µg/mL $^{13}C_3$ sodiumlactate solution) each, 1 µg of IS only, blank RPMI, and RPMI with 1 µg of IS.

1.2.1 Estimation of lactate blank values with calibration samples

The calibration samples had either no additional lactate weighed in, like blank RPMI medium, known amounts of unlabeled lactate or 1 µg of labeled lactate (IS). In addition, they carried a blank value of unknown size. Thus, the total amount of lactate [lac] in a calibration sample equals:

$$total\_amount = [lac]_{labeled} + [lac]_{unlabeled} + [lac]_{blank\ value}$$

Unlabeled and labeled values were known from sample preparation and the blank value had to be determined for each calibration sample. To convert the lactate amount into the lactate signal as registered by the MS, we implemented a conversion factor r.

$$signal = r * total\_amount$$

The measured CMD of a given calibration sample was set together by the fraction of the respective lactate origin times the CMD it contributes.

$$CMD_{calibration\ sample} = f_{blank\ value} * n^3 + f_{unlabeled} * n^3 + f_{labeled} * t^3$$

with the fractions being calculated from the total lactate amounts, e.g.:

$$f_{blind\ value} = \frac{[lac]_{blank\ value}}{total\_amount}$$

The evaluation of the calibration samples was based on the following requirement:

- Each calibration sample had its own $[lac]_{blank\ value}$ and conversion factor *r*. Their actual values should be a sample from a normal distribution, whose mean and standard deviation are determined from the set of calibration samples.
- Individual values for $[lac]_{blank\ value}$ and conversion factor *r* were adapted such that resulting calculated signal areas and $CMD_{calibration\ sample}$ matched the corresponding GC/MS measurements

From the calibration samples, the model could now estimate both the conversion factor r (median 628, IQR 298;876) and the average amount of blank value lactate (median 0.26 µg, IQR 0.12;0.36) across all calibration samples. We set the sampling statements so that the CMDs and total signals calculated with the sampled *r* and blank value parameters should come close to the ones measured by GC/MS.

1.2.2 Estimation of $Glyc_2$

After estimating the average blank value and the conversion factor, we could apply the same principle to the cell supernatants. The lactate sources now included the lactate secreted by the cells, which acted as one of the unknown parameters. Each sample was measured twice: once with and once without an additional 1 µg of IS, corresponding to a labeled lactate source.

$$signal = \mathrm{r} * ([lac]_{labeled} + [lac]_{blank} + [lac]_{secreted})$$

$$CMD_{sample} = f_{blank\ value} * n^3 + f_{labeled} * t^3 + f_{secreted} * CMD_{secreted}$$

As both samples originated from the same cell incubation, differing only in the addition of IS, $CMD_{secreted}$ should be the same for both measurements and was therefore sampled as such.

Sampling statements included:

- Individual blank values and conversion factors should stay in the range of their mean value and standard deviation as calculated with the calibration samples
- $CMD_{secreted}$ should be similar to the pyruvate labeling calculated in the primary model and should be the same for a sample measured with or without IS (^{13}C sodiumlactate)
- The calculated total signal should come close to the one measured with GC/MS
- The calculated CMD_{sample} should come close to the one measured with GC/MS

These sampling statements resulted in an estimated lactate amount released into the medium ($[lac]_{secreted}$), from which the lactate secretion $Glyc_2$ could be derived. $Glyc_2$ could be determined with a median standard deviation of ± 20.25 % (IQR 17.53 %; 22.75 %).

1.3 Tertiary model: Estimation of fluxes

When only tracer distributions are used, absolute flux rates cannot be determined. Accordingly, PPP fluxes could only be calculated in relation to a fixed Q_2 value, while in the TCA model only the relative fluxes R_1 to R_6 were estimated. However, it is possible to determine absolute flux rates from lactate released into the medium, corresponding to $Glyc_2$, and from the $^{13}CO_2$ production. The latter can be calculated as

$$^{13}CO_2\ \text{production} = F_2 pyr_1 + F_4 o_1 + F_3 akg_1 + F_5 o_4 + Q_3 h_1$$

where h_1, pyr_1, o_1, o_4, and akg_1 are CMDs for single carbons as estimated in the TCA model.

In the following, we scale the PPP values and transform the TCA cycle values so that we obtain a set of absolute fluxes for both models. With this set of parameters (i.e. fluxes), we should be able to calculate a combined lactate accumulation (as determined in 1.2.2.) and three different $^{13}CO_2$ productions corresponding to the measurements for each of the three respective tracer approaches.

For TCA cycle fluxes, we had the four balances of OAA, αKG, pyruvate and ACC. In consequence, the four fluxes F_6, F_8, L_{ACC} and F_9 could be calculated according to the equations below (deduced from Table S1), while F_2, F_3, F_4, F_5, F_7, F_{11}, L_{OAA}, L_{Pyr}, and $Glyc_2$ had to be sampled independently. $Glyc_1$ was calculated from the sampling of Q_2 and Q_{11}.

$$F_8 = F_3 - F_4$$

$$F_6 = Glyc_1 + F_5 + F_{11} - F_2 - L_{Pyr} - Glyc_2$$

$$L_{ACC} = F_2 + F_7 - F_4$$

$$F_9 = F_5 + F_4 + L_{OAA} - F_3 - F_6$$

$$Glyc_1 = 2Q_2 + Q_{11}$$

Q fluxes were estimated by firstly sampling a lactate production $Glyc_2$ that should come close to the value determined in 1.2.2. We further estimated a set of reference fluxes Q_x_ref (e.g. Q_5_ref or Q_{11}_ref) as random samples of the parameter distribution obtained in Section 1.1, while Q_2_ref was set to the fixed Q_2. We implemented a 'flux scaling" variable FS, which was used to convert fluxes from Q_3_ref to Q_3. FS was adapted such the resulting fluxes were in mass balance with $Glyc_2$ and was sampled as an unknown parameter (Table S1). Important parameters and their priors are listed in Table S5.

$$FS = \frac{2Q_2\_reference + Q_{11}\_reference}{2Q_2\_sampled + Q_{11}\_sampled}$$

Moreover, the PPP fluxes and TCA fluxes were sampled so that the ratios calculated from fluxes came close to the ones calculated from the first Stan run.

We had two additional measurements mentioned above that helped to characterize the fluxes: $^{13}CO_2$ production and lactate accumulation in the medium. Fluxes were be sampled so that the calculated $^{13}CO_2$ production came close to the one calculated in the first and secondary model.

2 Analyzing the informative content of aspartate and glutamate fragments

In Bayesian estimation, iterations of complete sets of parameters are collected in a sampling chain (section 1). From the collection of accepted samples, a 'posterior' can be determined for each parameter. In addition, each sample can be plotted in pairs, from which it can be seen to which extent the parameters/flux values correlate.

We used the sampling chain data to analyze how our measurement data impacts our parameter distributions to investigate whether our network is underdetermined. Figure S2 demonstrates the posterior data of flux ratios for T1 granulocytes of an exemplary animal. The pink histogram shows the prior distributions as obtained without fragment measurement input. The purple histogram displays

the posterior distribution of the same model only with inclusion of parameter fitting to fragment CMDs. The narrow distributions for r_3 and r_4 demonstrate how TCA cycle ratios can be accurately assessed with glutamate and aspartate fragments only. Figure S3 and S4 show the pairs plot of the respective model fits. Each point in the off-diagonal panels reflects a sample of the collected sampling chain. As a result of fitting to measurement data, the data in Figure S4 is presented with a much smaller scaling than in S3.

We further analyzed how the high ratio variance in the primary model impacts flux determination in the tertiary model. When estimating fluxes in the tertiary model, ratios and their standard deviation from the primary model are utilized as data input. We fit the tertiary model once for each set of ratios and their variance. The corresponding posterior distributions are displayed in Figure S5 and their pairs plots in Figure S6 and S7. Similarly, to the pairs plots of the primary model, the data points in Figure S7 are shown with a significantly smaller scaling than in S6.

3 Supplementary Figures and Tables

3.1 Supplementary Tables

Table S1: Input-Output relations of different metabolite pools.

Symbol	Metabolite	Input		Output
a	*ACC*	$F_2 + F_7$	=	$F_4 + L_{ACC}$
o	*OAA*	$F_3 + F_6 + F_9$	=	$F_5 + F_4 + L_{OAA}$
akg	*aKG*	$F_4 + F_8$	=	F_3
ci	*citrate*	F_4	=	F_4
suc	*succinate*	F_3	=	F_3
pyr	*pyruvate*	$Glyc_1 + F_5 + F_{11}$	=	$Glyc_2 + F_2 + F_6 + L_{Pyr}$
h	*hexose*	$Q_1 + Q_5 + Q_9$	=	$Q_2 + Q_3 + Q_6 + Q_{10}$
p	*pentose*	$Q_3 + Q_6 + 2Q_8$	=	$Q_4 + Q_5 + 2Q_7$
e	*erythrose*	$Q_6 + Q_9$	=	$Q_5 + Q_{10}$
s	*sedoheptulose*	$Q_7 + Q_{10}$	=	$Q_8 + Q_9$
tr	*triose*	$Q_5 + Q_7 + Q_{10}$	=	$Q_6 + Q_8 + Q_9 + Q_{11}$

Table S2: List of TBDMS derivatives of metabolite fragments as measured with GC/MS [10].

Metabolite	carbon	m/z
glutamate	c_1 - c_5	432 – 439
glutamate	c_2 - c_5	330 – 336
aspartate	c_1 - c_4	418 – 423
aspartate	c_2 - c_4	390 – 395

aspartate	c_1 - c_2	302 – 308
lactate	c_1 - c_3	260 – 264
lactate	c_2 - c_3	232 – 236

Table S3: End concentrations of important components in the three different RPMI media.

	**1,2-$^{13}C_2$ glucose medium**	**$^{13}C_6$ glucose medium**	**$^{13}C_5$ glutamine medium**
unlabeled glucose [g/L]	0.9	0.9	2
labeled glucose [g/L]	0.9	0.9	-
unlabeled glutamine [g/L]	0.6	0.6	-
labeled glutamine[g/L]	-	-	0.6
$NaHCO_3$ [g/L]	0.1	0.1	0.1
HEPES [g/L]	-	-	-
RPMI stock (Sigma Aldrich, St. Louis, MO, USA)	RPMI-1640 R1383		RPMI-1640 R1145 + 1 mg/L folic acid

Table S4: Important primary model parameters and their priors. Lowercase letters indicate transformed parameters. stdPrior = 0.3

**parameter**	**parameter bounds**	**model prior**	**transformation**
r_1	<lower=−7,upper=7>	~ normal(ln(0.9), 8*stdPrior)	ln(x/(1−x))
r_2	<lower=−7,upper=0>	~ normal(ln(0.84), 3*stdPrior)	ln(x)
r_3	<lower=−7,upper=0>	~ normal(ln(0.7), 3*stdPrior)	ln(x)
r_4	<lower=−7,upper=0>	~ normal(ln(0.5), 3*stdPrior)	ln(x)
r_5	<lower=−7,upper=0>	~ normal(ln(0.03), 3*stdPrior)	ln(x)
r_6	<lower=−7,upper=0>	~ normal(ln(0.02), 3*stdPrior)	ln(x)
v	<upper=0.0>	~ normal(−0.41, 3*stdPrior)	ln(x)
k	<upper=0.0>	~ normal(−0.51, 3*stdPrior)	ln(x)
w	<upper=0.0>	~ normal(−0.41, 3*stdPrior)	ln(x)
z	<lower=−7,upper=7>	~ normal(0, 6*stdPrior)	ln(x/(1−x))

Table S5: Important tertiary model parameters and their priors.

parameter	parameter bounds	model prior
F_2	<lower=0.025,upper=200>	~ normal(5, 4)
F_4	<lower=0.025,upper=200>	~ normal(5, 4)
F_5	<lower=0.025,upper=200>	~ normal(5, 4)
Q_4	<lower=0.025,upper=200>	~ normal(15, 15)
Q_5	<lower=0.025,upper=400>	~ normal(15, 15)

3.2 Supplementary Figures

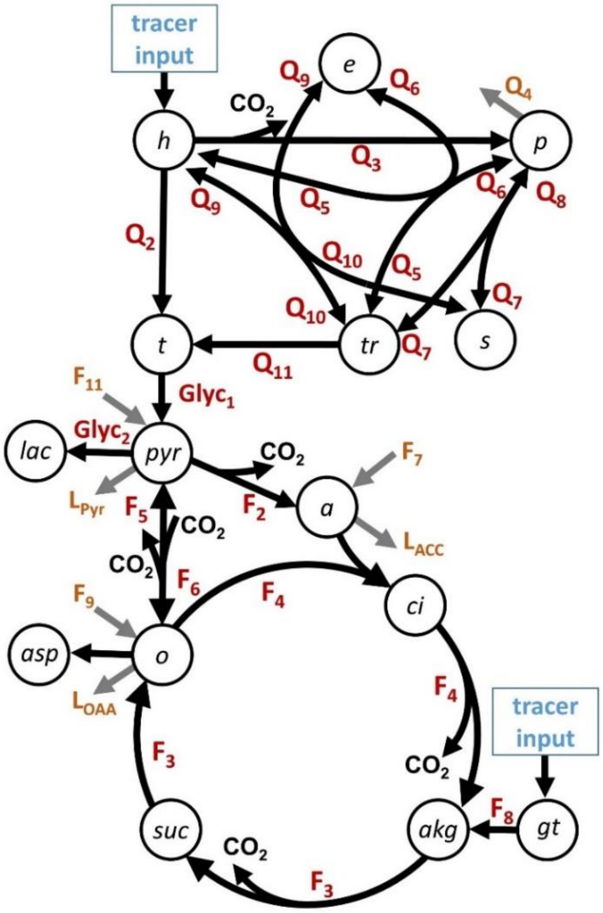

Figure S1. Visualization of the model used for Bayesian sampling. Glyc: glycolytic flux. Q: flux within the PPP. F: flux within the TCA cycle. L: flux leaving the network. Abbreviations are explained in Table S1.

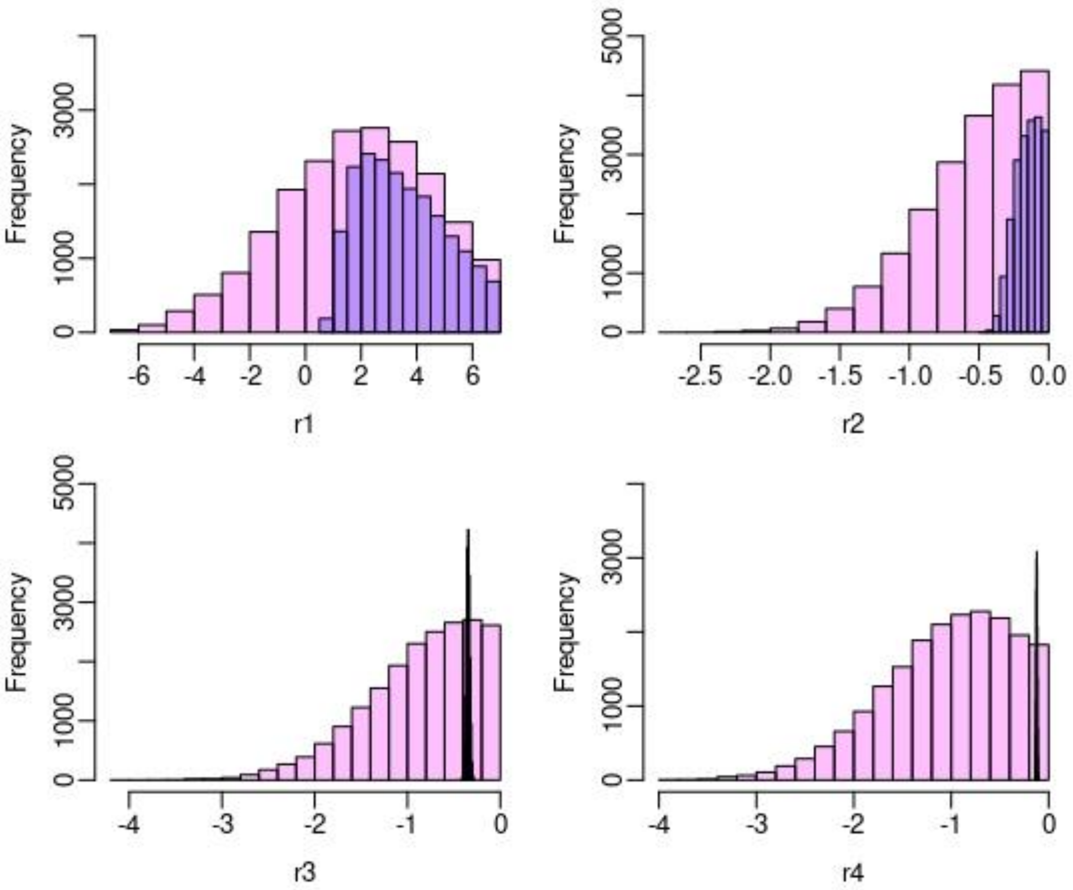

Figure S2. Prior and posterior distributions of selected parameters in the primary model. Methods for parameter transformation and parameter bounds are specified in Table S4. The pink histogram shows the prior distribution resulting from no fitting to measurement data. The purple histogram shows the posterior distribution with fitting to measurement data.

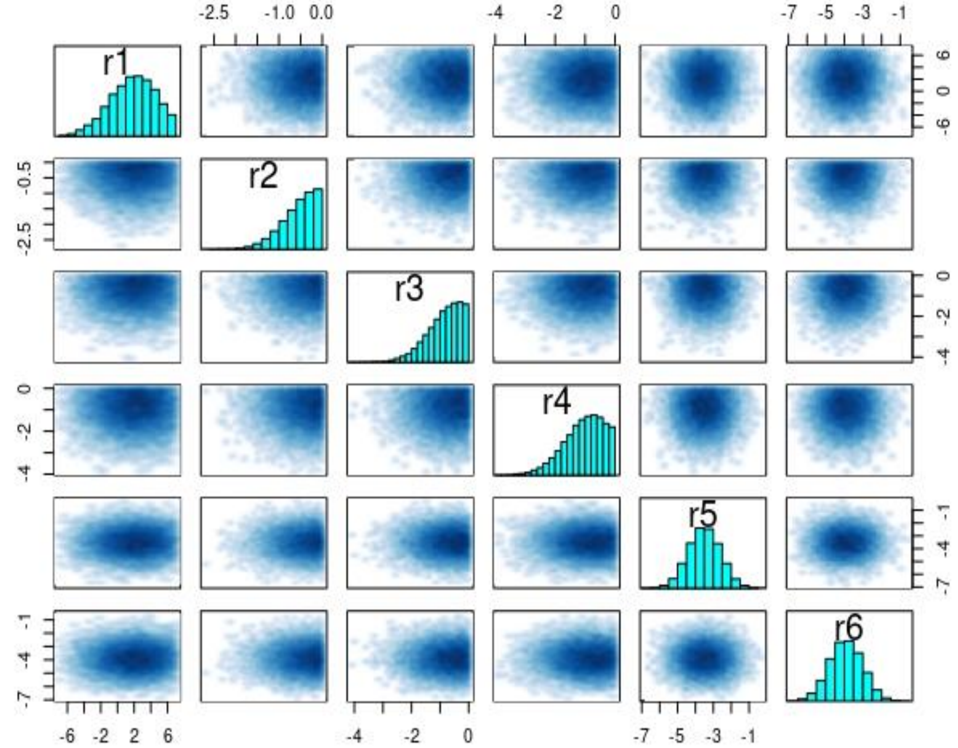

Figure S3. Pairs plot of ratios in the primary model. Individual data points (blue) are sampled values of prior distributions. Parameters were not fitted to measurement data.

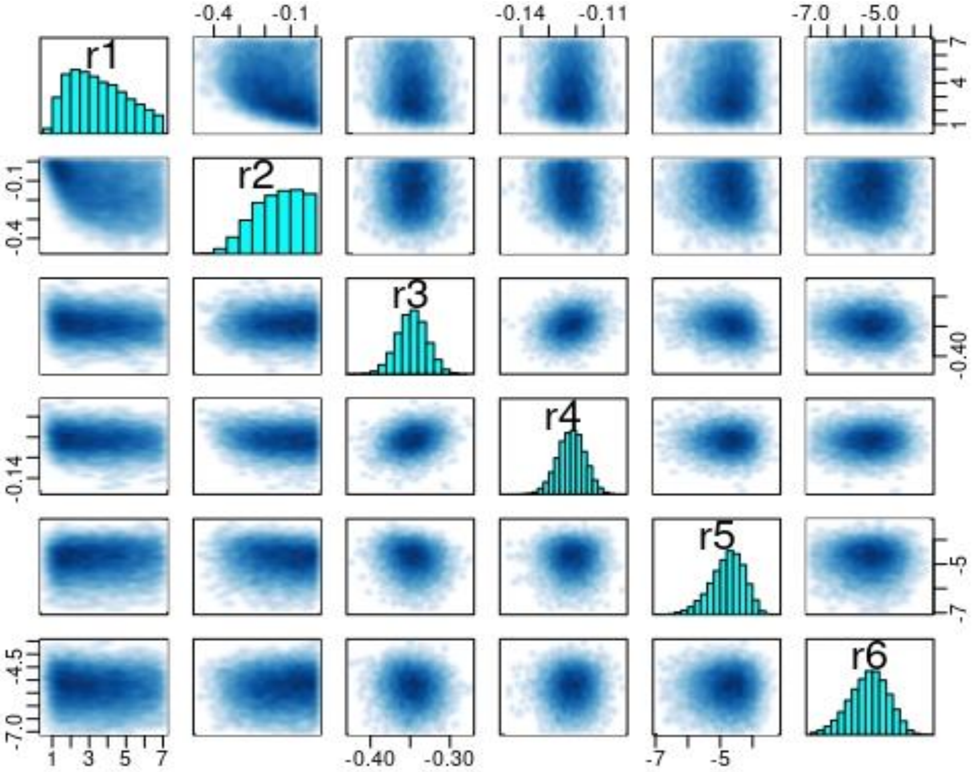

Figure S4. Pairs plot of ratios in the primary model. Individual data points (blue) are sampled values of posterior distributions. Parameters were fitted to measurement data.

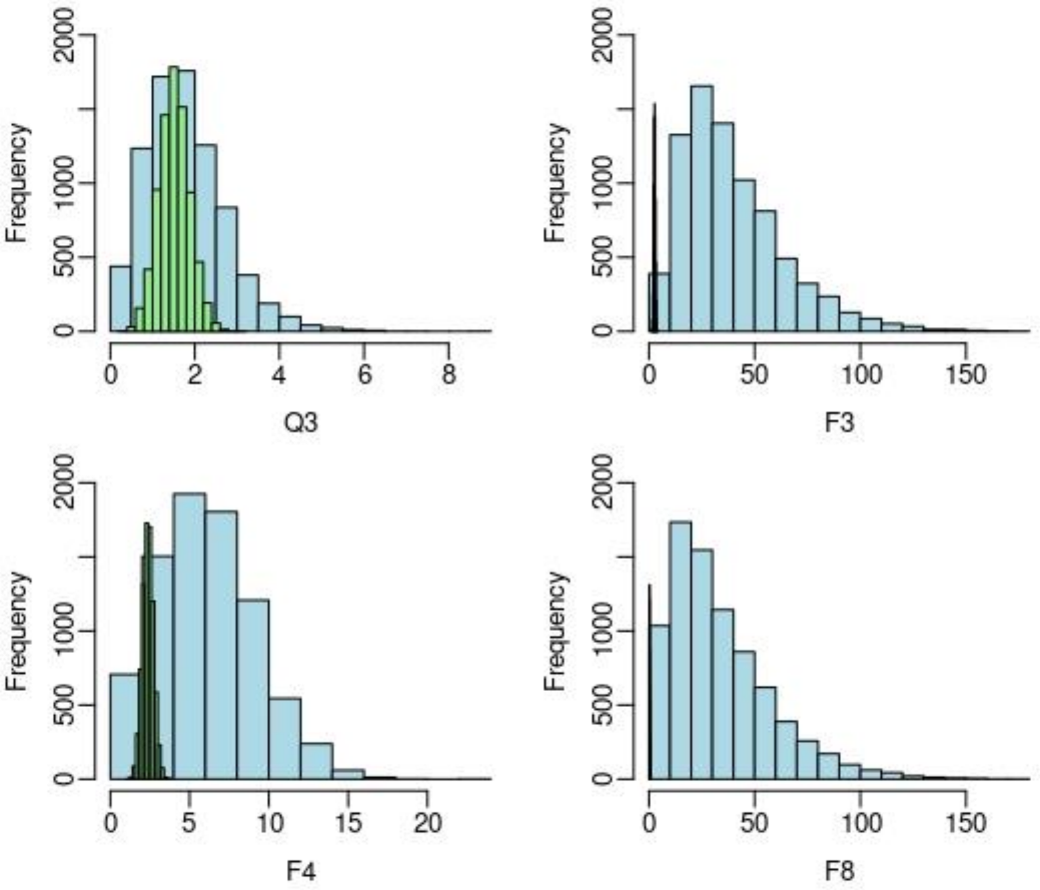

Figure S5. Prior and posterior distributions of selected parameters in the tertiary model. The blue histogram shows the prior distribution of parameters resulting from no fitting to measurement data in the primary model. The green histogram shows the posterior distribution of parameters in case fitting to measurement data was included in the primary model.

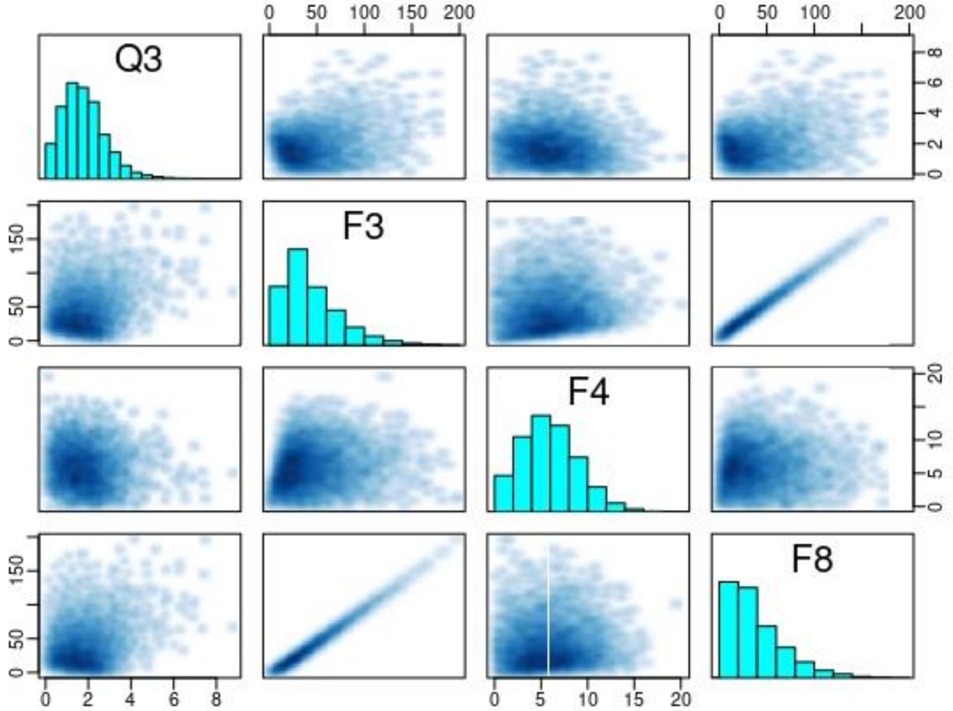

Figure S6. Pairs plot of fluxes in the tertiary model. Individual data points (blue) are sampled values of prior distributions. Parameters were not fitted to measurement data.

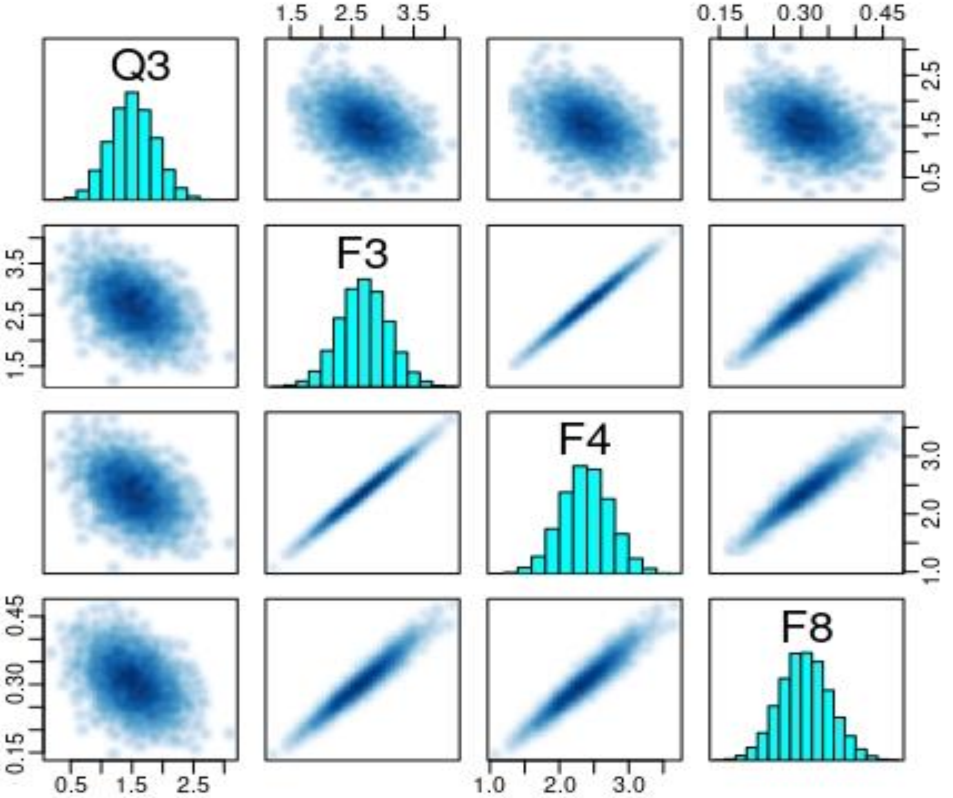

Figure S7. Pairs plot of fluxes in the tertiary model. Individual data points (blue) are sampled values of posterior distributions. Parameters were fitted to measurement data.

4 REFERENCES

1. Stan Development Team. RStan: the R interface to Stan (2020). Available from: http://mc-stan.org/
2. Weitzel M, Nöh K, Dalman T, Niedenführ S, Stute B, Wiechert W. 13CFLUX2--high-performance software suite for (13)C-metabolic flux analysis. *Bioinformatics* (2013) **29**:143–5. doi:10.1093/bioinformatics/bts646
3. Alger JR, Sherry AD, Malloy CR. tcaSIM: A Simulation Program for Optimal Design of 13C Tracer Experiments for Analysis of Metabolic Flux by NMR and Mass Spectroscopy. *Curr Metabolomics* (2018) **6**:176–87. doi:10.2174/2213235X07666181219115856
4. Antoniewicz MR, Kelleher JK, Stephanopoulos G. Elementary metabolite units (EMU): a novel framework for modeling isotopic distributions. *Metab Eng* (2007) **9**:68–86. doi:10.1016/j.ymben.2006.09.001.
5. Wiechert W. 13C metabolic flux analysis. *Metab Eng* (2001) **3**:195–206. doi:10.1006/mben.2001.0187.
6. Vogt JA, Yarmush DM, Yu YM, Zupke C, Fischman AJ, Tompkins RG, et al. TCA cycle flux estimates from NMR- and GC-MS-determined 13Cglutamate isotopomers in liver. *Am J Physiol* (1997) **272**:C2049-62. doi:10.1152/ajpcell.1997.272.6.C2049
7. Lee WN, Boros LG, Puigjaner J, Bassilian S, Lim S, Cascante M. Mass isotopomer study of the nonoxidative pathways of the pentose cycle with 1,2-13C2glucose. *Am J Physiol* (1998) **274**:E843-51. doi:10.1152/ajpendo.1998.274.5.E843
8. Katz J, Rognstad R. The labeling of pentose phosphate from glucose-14C and estimation of the rates of transaldolase, transketolase, the contribution of the pentose cycle, and ribose phosphate synthesis. *Biochemistry* (1967) **6**:2227–47. doi:10.1021/bi00859a046
9. Malloy CR, Sherry AD, Jeffrey FM. Evaluation of carbon flux and substrate selection through alternate pathways involving the citric acid cycle of the heart by 13C NMR spectroscopy. *J Biol Chem* (1988) **263**:6964–71.
